# Promoting active outdoor play and healthy dietary behaviours through the co-creation of supporting physical and social environments for and with primary school-aged children living in underserved neighbourhoods in Europe: the protocol of the B-Challenged project

**DOI:** 10.1136/bmjopen-2025-108281

**Published:** 2026-03-06

**Authors:** Teatske M Altenburg, Charlotte S Pawlowski, Wolfgang Ahrens, Tilman Brand, Christoph Buck, Claudia Börnhorst, Anna Dzielska, Leonie Klaufus, Pilar De Miguel-Etayo, Luis Moreno, Katarzyna Okulicz-Kozaryn, Tanja G M Vrijkotte, Renée Wink, Laura S Belmon, Mai J M Chinapaw

**Affiliations:** 1 Public and Occupational Health, Amsterdam UMC Locatie AMC, Amsterdam, Noord-Holland, Netherlands; 2 Health Behaviours and Chronic Disease, Amsterdam Public Health Research Institute, Amsterdam, Netherlands; 3 Methodology, Amsterdam Public Health Research Institute, Amsterdam, Netherlands; 4 University of Southern Denmark, University of Southern Denmark, Odense, Denmark; 5 Epidemiological Methods and Etiological Research, Leibniz-Institut fur Praventionsforschung und Epidemiologie - BIPS GmbH, Bremen, Bremen, Germany; 6 Leibniz-Institut fur Praventionsforschung und Epidemiologie - BIPS GmbH, Bremen, Bremen, Germany; 7 Institute of Mother and Child Department of Child and Adolescent Health, Warsaw, Masovian Voivodeship, Poland; 8 Public and Occupational Health, Amsterdam UMC, location Vrije Universiteit Amsterdam, Amsterdam, Noord-Holland, Netherlands; 9 Growth, Exercise, Nutrition and Development (GENUD-B34_23R) Research Group; Instituto Agroalimentario de Aragón (IA2), Instituto de Investigación Sanitaria Aragón (IIS Aragón), University of Zaragoza, Zaragoza, Spain; 10 Centro de Investigación Biomédica en Red de Fisiopatología de la Obesidad y Nutrición (CIBERObn), University of Zaragoza, Zaragoza, Spain; 11 Public and Occupational Health, Amsterdam UMC, location University of Amsterdam, Amsterdam, Netherlands

**Keywords:** Community-Based Participatory Research, PUBLIC HEALTH, Primary Prevention

## Abstract

**Introduction:**

An alarmingly low number of children meet public health guidelines for physical activity and dietary behaviours and, therefore, are at increased risk of developing lifestyle-related diseases. This paper describes the protocol of the B-Challenged project, which aims to co-create systemic actions to promote active outdoor play and healthy dietary behaviours before, during or after their outdoor play together with children themselves.

**Methods and analysis:**

In five European countries, child-centred Participatory Action Research (PAR)—combined with systems dynamics methods—was conducted with 15–20 child co-researchers (aged 9–12 years) and 15–20 adult actors (eg, youth workers, local policy makers). In the first phase, the main drivers of children’s active outdoor play and related dietary behaviours were mapped by (1) analysing existing cohort data, and (2) conducting child-centred PAR. In the second phase, systemic actions targeting the local physical and social environments will be co-created and implemented by child co-researchers and adult actors to promote children’s active outdoor play and related healthy dietary behaviours. A mixed-methods design will be used to evaluate (1) if actions positively contributed to systems change and 6- to 12-year-olds’ outdoor play and related dietary behaviours (140 children per country); (2) the process of conducting multi-actor, child-centred PAR and implementing the co-created actions and (3) if the child-centred PAR positively contributed to child co-researchers’ feelings of empowerment.

**Ethics and dissemination:**

Ethics approval for the mapping phase was obtained and approval for implementation and evaluation will be obtained from the five local research institutions. Participating children, one of their parents/caregivers and adult actors had given informed consent before participating in the project. Throughout the project, child-friendly methods, materials and language will be applied, and ethical challenges and potential solutions will be discussed. Project results will be disseminated locally and internationally through various channels and activities among the scientific community, professionals—for example, in health and policy making, children and other citizens.

**Trial registration number:**

NCT07136376.

**STRENGTHS AND LIMITATIONS OF THIS STUDY**

- By combining causal inference analyses of cohort and area-level monitoring data with a child-centred Participatory Action Research (PAR) and systems dynamic approach, B-Challenged will provide important insights into the complex mechanism driving children’s behaviours.
- The application of child-friendly methods and adoption of child-friendly terminology will enable child and adult actors to understand relevant concepts and contribute actively and meaningfully throughout the process of co-creating and implementing systemic actions.
- Including both child co-researchers and relevant adult actors throughout the child-centred PAR process on a voluntary basis is challenging and poses risks of declining participation or dropping out.
- The longitudinal cohort analysis with adjacent age cohorts will allow for a controlled design without the need to newly recruit schools and children serving as controls.
- The reliance of the effect evaluation on self-report data may introduce recall and social desirability biases.

## Introduction

 An alarmingly low number of children and adolescents meet public health guidelines on physical activity and healthy dietary behaviours: only 7%–33% of 5- to 17-year-old children and adolescents globally are sufficiently active,^1^ 4%–52% of European children and adolescents meet European guidelines for fruit and vegetable intake^2^,^4^ and, for example, more than half of 12- to 17-year-old German adolescents do not achieve German food-based dietary recommendations for fruits and vegetables, and consume far above the recommended amounts of unfavourable foods such as confectionery.^5^ Consequently, these children have a higher risk of lifestyle-related non-communicable diseases.^6^ Importantly, this burden is disproportionately distributed across educational, employment and migrant groups, with children of parents with low/medium (vs high) education, children with unemployed (vs employed) parents and children with a migrant background (vs native) being less likely to engage in favourable health-related behaviours resulting in higher proportions of overweight.^7^,^9^

For children, outdoor play is an important source for their moderate-to-vigorous physical activity.^10^ However, only 47%–53% of children across 21 European countries meet the recommendation of actively playing (including playing outdoors) for at least 2 hours per day at any intensity.^1^ Importantly, children living in underserved neighbourhoods play outside less than their peers growing up in more affluent neighbourhoods,^11^,^13^ with underserved neighbourhoods defined in this study as neighbourhoods with a high percentage of families characterised by lower income, lower levels of education, more health disadvantages (eg, physical/mental health problems, safety issues) and limited availability of attractive and safe (green) spaces for outdoor play. Reasons for this include that children living in underserved neighbourhoods have less access to safe and well-maintained outdoor play spaces and appropriate and affordable after-school activities compared with their peers in more affluent neighbourhoods^14^,^17^ and their mothers are more hesitant to stimulate their children’s outdoor play because of crime and safety concerns.^18 19^

Concurrently, today’s children have easy access to inexpensive energy-dense foods^20 21^ and outdoor advertising of unhealthy foods and beverages is located near places children frequently visit such as schools and playgrounds,^22^ particularly in underserved neighbourhoods.^16 23 24^ As most children have a preference for energy-dense foods and drinks, are sensitive to external food cues and experience difficulties with matching intake to physiological energy demands,^14 16 25^ it is important that outdoor spaces promote healthy dietary behaviours (eg, through water fountains) and limit access to unhealthy foods and beverages.

Previous interventions aimed at promoting favourable health-related behaviours in children had only small and short-term effects, especially among those from underserved neighbourhoods.^26 27^ Consequently, health inequalities tend to be maintained or even widened.^28 29^ One explanation for this is that most interventions do not take into account the complexity of the various, multidimensional and interconnected factors that drive public health problems such as unfavourable health-related behaviours in children.^16 30^ Approaches from systems dynamics are aimed at tackling complex problems by addressing the dynamic nature and interconnectedness between the various factors within a system that drive complex problems,^31^ and are therefore increasingly used to obtain a better understanding of complex problems such as unfavourable health-related behaviours, and subsequently develop actions to change the system.^32 33^ For example, when the neighbourhood is perceived as unsafe by children, they perceive the environment as less attractive, which results in children playing less outdoors, weakening normalisation of active outdoor play and active commuting to school.^34^ At the same time, access to unhealthy snacks and exposure to unhealthy snack marketing leads to peer pressure to eat unhealthy snacks, subsequently leading to a normalisation of unhealthy snacks.^35^

A second explanation for the limited effectiveness of interventions is that the perspectives of children themselves are rarely considered when developing interventions. Instead, in child-centred Participatory Action Research (PAR) children are actively involved in an iterative research process that is aimed at generating knowledge-for-action and knowledge-through-action.^36 37^ As co-researchers, children conduct peer research and collaborate with key adult actors to ensure that the identified problems and potential solutions are supported by their peers and other key actors. Through the central concept of co-learning, children learn about conducting research, evidence-based practice and initiating change. In addition, (academic) researchers learn about the lived experiences of children, that is, gaining a deeper understanding of the problems under study. A recent systematic review summarising the impact of participatory approaches to develop and implement interventions targeting physical activity and sedentary behaviour showed that such approaches resulted in children’s feelings of empowerment, experiences of ownership and gaining skills such as decision-making and leadership.^38^

This paper describes the design of the B-Challenged project, combining child-centred PAR with methods from systems dynamics to co-create an action programme aimed at promoting active outdoor play and related healthy dietary behaviours for and with children growing up in underserved urban neighbourhoods in five European countries (Denmark, Germany, Poland, Spain and the Netherlands). With related healthy dietary behaviours, we refer to children’s consumption of water, fruits and vegetables right before, during or following their outdoor play. Additionally, this paper describes the evaluation design of a mixed-methods approach to evaluate (1) if the co-created and implemented actions positively contributed to systems change (eg, local policy makers and urban planners sustainably engage children in the development of attractive playgrounds) and children’s outdoor play and related healthy dietary behaviours; and (2) the process of conducting child-centred PAR and implementing the co-created systemic actions and (3) if the child-centred PAR process positively impacted child co-researchers’ feelings of empowerment.

## Methods

### Study design

The B-Challenged approach of child-centred PAR with methods from systems dynamics will be applied in two phases: (1) mapping the main drivers of children’s outdoor play and related dietary behaviours in the physical and social environment, through cohort analysis (phase 1A) and child-centred PAR (phase 1B); and (2) co-creation and implementation of systemic actions targeting the physical and social environment to improve children’s active outdoor play and related healthy dietary behaviours. In each country, a practical protocol for applying child-centred PAR with systems dynamics methods will be followed, involving multiple actors (child co-researchers and adult actors) in the co-creation and implementation of actions targeting children’s active outdoor play and related dietary behaviours. This protocol was based on the Youth-centred Participatory Action (YoPA) co-creation protocol^39^ and describes the general outline of the child-centred PAR approach, through which children are actively involved as co-researchers. The protocol included proposed activities, while allowing the flexibility to adapt sessions to the local context, and child-friendly wordings of theoretical concepts. Both the B-Challenged and YoPA protocols were based on the five building blocks of Cornish^37^ to conduct PAR: building relationships, establishing working practices, establishing a common understanding of the problem, observing, gathering and generating materials and collaborative analysis. Age-appropriate participatory methods were used in the sessions with children, such as photovoice.^40^ Capacity building was an important aspect of the child-centred PAR approach, to enable child co-researchers to conduct research (eg, research methods, privacy protection), conduct basic analyses of collected data and present findings, for example, by presenting various research methods, facilitating discussions on how to protect privacy, demonstrating how to analyse data and practicing presentations. Additionally, and similar to the YoPA co-creation protocol,^39^ this protocol was based on methods from systems dynamics: (1) Group Model Building,^41^ to develop a shared understanding of the main factors and interrelations that drive children’s active outdoor play and dietary behaviours in the local neighbourhoods, and (2) the Action Scales Model,^42^ to describe the four levels at which action could take place, with increasing impact on system change (ie, events, structures, goals and beliefs). Facilitating researchers in each country applied the practical protocol to their local context. Monthly reflection meetings were organised to support facilitators and align the child-centred PAR process across countries.

**Figure 1:**
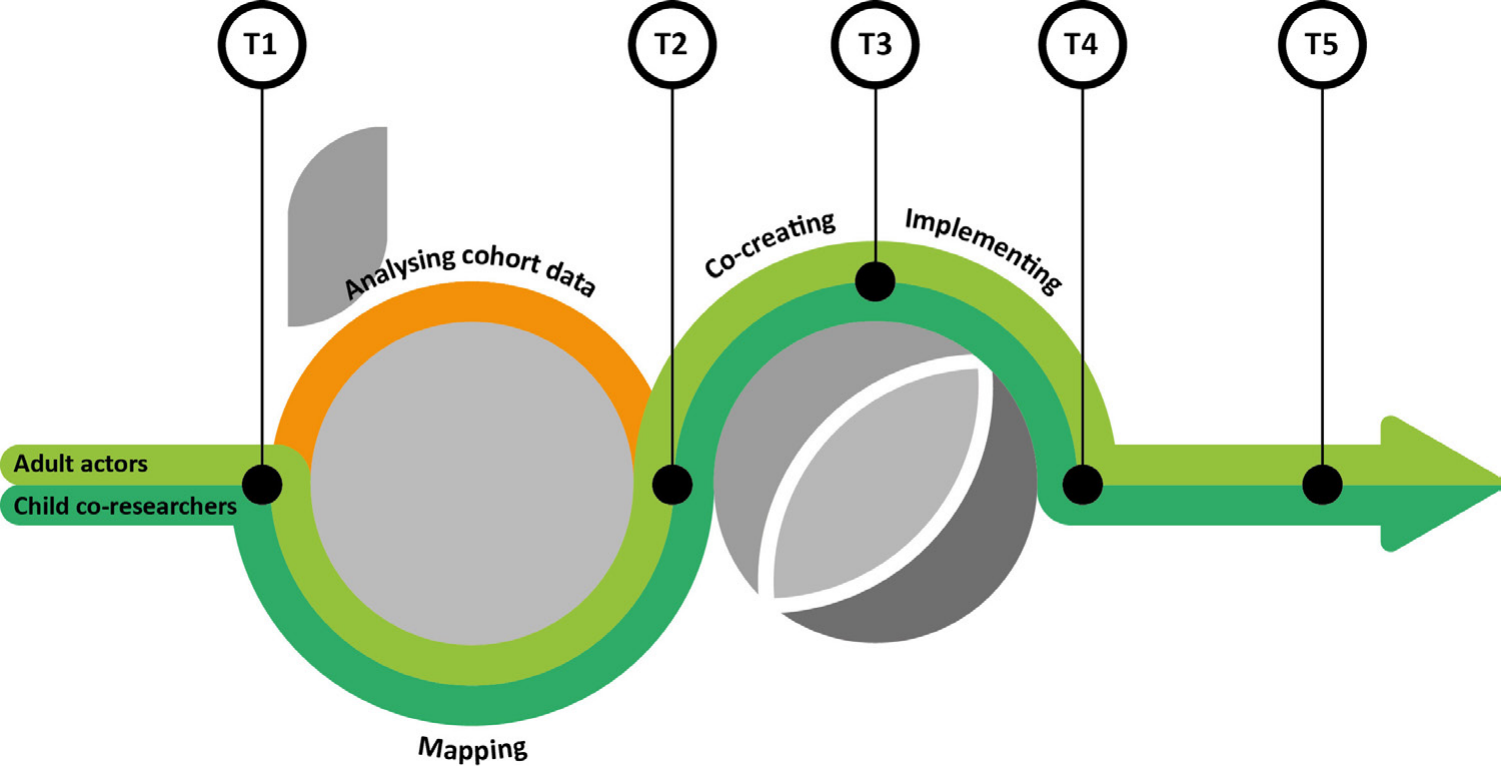
Design of the B-Challenged project, including analysis of cohort data (orange) and Participatory Action Research with child co-researchers and adult actors (green). T1–T5 represent specific evaluation time points: T1, at the start of the mapping phase; T2, immediately after the mapping phase; T3, before implementation of co-created actions; T4, after implementation of co-created actions; T5, approximately 6 months after T4. Process evaluation occurs continuously between T1 and T5 (eg, facilitators’ logbooks, reflection forms).

Through a mixed-methods approach, we will evaluate whether co-created and implemented actions positively contributed to system change and children’s outdoor play and related dietary behaviours. Additionally, we will evaluate the process of conducting child-centred PAR and the process of implementing co-created actions. The B-Challenged project started in May 2024 and will continue until April 2027. Figure 1 visualises the design of the B-Challenged project, including the different phases and the evaluation time points.

### Setting and participants

The B-Challenged project aims to stimulate active outdoor play and healthy dietary behaviours in primary school-aged children—aged 6–12 years—living in underserved neighbourhoods in the five European countries. In each country, one underserved urban neighbourhood was selected, based on our definition of having a high percentage of families characterised by lower income, lower levels of education, more health disadvantages (eg, physical/mental health problems, safety issues) and limited availability of attractive and safe (green) spaces for outdoor play. Online supplemental file 1 provides descriptions of the sociodemographic and physical environment of the selected neighbourhoods in each participating country. B-Challenged focuses on primary school-aged children as (1) childhood is a critical period to establish healthy physical activity and dietary behaviours^43^; (2) children in this age group start to play outside independently, that is, without adult supervision^44^ and (3) children in this age group often start to receive pocket money,^45^ which allows them to buy unhealthy foods.^16 46^

#### Children participating as co-researchers in the co-creation and implementation of actions

In previous research, we demonstrated that children from the age of 9 years have an increased attention span, can more clearly see the viewpoint of others, are capable of participating in research projects, when using participatory age-appropriate methods, and are able to think for their younger peers when mapping needs and co-creating actions.^47 48^ Therefore, in each local neighbourhood, 15–20 children aged 9–12 years were recruited to participate as co-researchers, representing 6- to 12-year-old children in their local neighbourhood. Children were recruited through schools and community organisations (eg, library, after-school care). Online supplemental file 1 provides a description of the process of selecting children for each selected neighbourhood. In case children drop out, for example, when moving to another school/neighbourhood or starting secondary school, additional child co-researchers will be recruited.

#### Key adult actors participating in the co-creation and implementation of actions

In each local neighbourhood, a network of 15–20 local adult actors, including parents, teachers, youth workers, academic researchers, urban planners, policy makers, was established through actor mapping,^49 50^ which was conducted by 1–2 members of the local research team. First, researchers generated a list of potentially relevant actors across various sectors (eg, education, urban planning, social services, sports), based on actors/organisations situated in the local neighbourhood who/that are expected to hold important perspectives and position regarding children’s outdoor play and dietary behaviours in the selected neighbourhoods. Next, interviews with the identified actors were conducted to define the position of the actors within the local context, through identifying the actors’ characteristics related to their position in the organisation, interest in the issue, power and leadership. In case adult actors drop out, for example, when getting another job, additional relevant adults will be recruited.

#### Children participating in the effect evaluation

For the effect evaluation, 140 children aged 6–12 years will be recruited through schools in each local neighbourhood. Considering a power of 80%, a significance level of 0.05 and a drop-out rate of 25%, this number of children will be needed in each neighbourhood to detect a small effect (d=0.3), corresponding to approximately 12 min/day of outdoor play.

### Phase 1: mapping the main drivers of children’s active outdoor play and related dietary behaviours

The main drivers of children’s active outdoor play and related dietary behaviours will be investigated through analysis of existing cohort data (phase 1A) and co-creation of local system maps together with child co-researchers and adult actors that visualise the main drivers of active outdoor play and related dietary behaviours (phase 1B). For the cohort analyses, data include physical and social environmental factors as well as area-level monitoring data linked to the IDEFICS/I.Family cohort (IDEFICS—Identification and prevention of Dietary- and lifestyle-induced health EFfects In Children and infantS; and I.Family study^51^) and the ABCD (Amsterdam Born Children and their Development^52^) cohort (phase 1A). The IDEFICS/I.Family and ABCD cohort studies will be selected as they include children from similar European countries as those included in the co-creation (in phase 1B and phase 2). Only waves including data of similarly aged children will be considered in the analyses. The results from the cohort analysis provide insights into the causality and interconnectedness of environmental factors influencing health behaviours in children and adolescents from diverse European regions. Evidence will subsequently be fed into the system maps that will be developed in phase 1B.

#### Phase 1A: cohort analyses

IDEFICS/I.Family is a European, multicentre population-based cohort study investigating the causes of diet- and lifestyle-related diseases in children, adolescents and their families. The baseline examination was conducted in 2007/2008 in 16 230 children aged 2–9 years from Belgium, Cyprus, Estonia, Germany, Hungary, Italy, Spain and Sweden. Follow-up surveys were conducted after 2, 6 and 13 years (ie, 2009/10, 2013/14 and 2020/21). Details are given in Ahrens *et al*.^51^ The ABCD study is a cohort of children born in Amsterdam, the Netherlands, who were followed up since pregnancy. The aim of ABCD is to investigate the effects of perinatal factors on health outcomes up to young adulthood with specific interest in ethnic and social disparities. Between January 2003 and March 2004, 8266 pregnant women in Amsterdam filled out the pregnancy questionnaire, of which 7050 (85%) gave permission for follow-up. The subsequent examination waves took place at around 5, 11, 15, 17 and 19 years of age. Details are provided in van Eijsden *et al*.^52^

Cohort data have been further enriched with physical and social environmental characteristics and area-level monitoring data.^53 54^ Residential addresses of participants were geocoded and procedures were developed to derive environmental exposures such as greenness from remote sensing data or walkability characteristics from open-source spatial data. Based on previous research^55 56^ and using the enriched data of the IDEFICS/I.Family and ABCD cohorts, we aim to answer the following research questions:

1. What would happen to children’s physical activity (eg, active commuting or playing outdoors) if we were to increase the walkability, bikeability and greenness within the neighbourhood they live in?
2. What would happen to children’s physical activity if a green space or playground would be available within the neighbourhood children live in?
3. What would happen to children’s intake of sweet and fatty foods and appetite if we were to increase greenness within the neighbourhood they live in?

Methods of causal inference such as g-computation will be used to estimate the effects of the above-mentioned hypothetical interventions based on our observational data,^57^ as randomised controlled trials to study the long-term effects of physical and social environmental exposures on health behaviours are typically impractical or unethical. Since the above-stated associations were found to be moderated by deprivation,^58^ we will further investigate the moderating effect of area-level and individual social position on the longitudinal associations using linear mixed models.

#### Phase 1B: understanding drivers of children’s active outdoor play and related dietary behaviours

Approximately 14–18 sessions with child co-researchers and 3–4 sessions with adult actors will be organised to identify drivers of children’s active outdoor play and related dietary behaviours.

Local system maps (storyboards in child-friendly wording) will be co-created using a child-friendly application of Group Model Building, a systems dynamics method to engage researchers and key community actors to collectively develop a shared understanding of the complex system that drives the community issue.^41^ The resulting system map visually represents the main factors and interrelations that drive the community issue. Age-appropriate participatory methods in this phase include, for example, photovoice,^40^ storytelling^59^ and neighbourhood asset mapping^60^ activities.

First, separate participatory sessions with child co-researchers and adult actors will be organised to identify multi-actor perspectives on enabling and hindering factors of children’s active outdoor play and related healthy dietary behaviours. Sessions with child co-researchers will be focused first on building trust and establishing team/working agreements. Thereafter, they will be focused on their own behaviour and subsequently children conduct peer research to collect the perspectives of their peers. Sessions with adult actors will be focused first on building trust and establishing a network, and subsequently on identifying their perspectives on children’s behaviours. Second, separate participatory sessions with child co-researchers and adult actors will be organised to develop system maps, explaining how the previously identified factors influence children’s active outdoor play and related dietary behaviours, focusing on the interconnections of identified variables. Third, participatory sessions with child co-researchers will be organised to combine the co-created system maps of child co-researchers and adult actors into one overall system map. Additionally, findings from the cohort analyses (phase 1A) will be integrated into this overall system map. Finally, one collaborative participatory session with both child co-researchers and key adult actors will be organised to get children’s and adults’ perspectives on added cohort findings, to co-create a shared understanding of the drivers of children’s active outdoor play and related dietary behaviours, and decide on priorities of what needs to be changed in the physical and social environment to stimulate children’s active outdoor play and healthy dietary behaviours.

### Phase 2: co-creating and implementing systemic actions to stimulate children’s active outdoor play and related dietary behaviours

Approximately 11–14 sessions with child co-researchers and 3–5 sessions with adult actors will be organised to co-create systemic actions, develop implementation plans and implement the co-created actions. The system maps developed in phase 1 will inform the co-creation of systemic actions. The protocol for phase 2 was based on the ‘planning and taking action’ building block of Cornish^37^ and the Action Scales Model,^42^ describing four levels (or leverage points) to intervene within a system, with increasing expected impact on system change:

1. Events—behaviours and outcomes that can be observed.
2. Structures—structures/patterns that cause the events.
3. Goals—goals/targets/ambitions that the (sub)system is working to achieve.
4. Beliefs—beliefs/norms/attitudes/values of individuals and organisations in the system.

Age-appropriate participatory activities in this phase include, for example, reversed brainstorming and role-playing activities.

First, separate participatory sessions with child co-researchers and adult actors will be organised to decide on which systems levels to intervene within the local system, taking the developed overall system map as a starting point. One collaborative session will be organised to prioritise these systems levels and identify key actors that have (decision-making) power and interest to intervene on the prioritised systems levels. As recommended by Nobles *et al*,^42^ intervening with several mutually reinforcing actions at different systems levels will be encouraged. Second, the academic research team will develop a theory of change, describing how intervening on the prioritised systems levels can bring about changes in the local system that are required to structurally improve children’s active outdoor play and related healthy dietary behaviours. This theory of change will be presented to the child co-researchers and adult actors for feedback and improvement. Third, participatory sessions with child co-researchers will be organised to co-create actions that align with the theory of change. Fourth, participatory sessions with child co-researchers will be organised to develop an implementation plan for the co-created actions. Where relevant, key adult actors will be invited to join the sessions in the third and fourth step, for example, to provide feedback on action ideas, co-create actions and implementation plans. Finally, the co-created actions will be implemented according to the implementation plan.

### Process and effect evaluation

The primary aim of evaluating the B-Challenged project is to investigate if actions were co-created and implemented at various systems level, if these actions had positively contributed to (local) system change and if these actions had contributed to changes in 6- to 12-year-old children’s outdoor play and dietary behaviours before, during or after their outdoor play. Second, we aim to gain a deeper understanding of how to best apply a PAR approach with both children and relevant adult actors in the different process stages and thus identify areas of improvement during the process (ie, formative evaluation), and third, to understand how the process of PAR had contributed to changes in 6- to 12-year-old children’s outdoor play and related dietary behaviours. Fourth, we aim to identify if the child-centred PAR process had a positive impact on the child co-researchers’ feelings of empowerment. Evaluating all stages in the process is important to identify all mechanisms that can explain how and why the child-centred PAR approach and co-created actions worked (or not), and under what conditions.^61^

The process and effect evaluation was informed by the previously developed SUPER-AIM evaluation protocol, which stands for: Systems, User perspectives, Participatory co-creation process, Effectiveness, Reach, Adoption, Implementation and Maintenance,^39^ inspired from the well-known and often used RE-AIM (Reach, Effectiveness, Adoption, Implementation, and Maintenance) framework.^62^ Each of the eight SUPER-AIM components covers several outcome items and will be evaluated using quantitative (eg, questionnaire, evaluation forms and systematic observation) as well as qualitative (eg, interviews and logbook) data sources. The evaluation will involve all partners participating in the co-creation (ie, child co-researchers, adult actors and academic facilitators), and a sample of children (6–12 years old) living in the five neighbourhoods.

Table 1 provides an overview of the SUPER-AIM components, outcome items, data sources and timepoints for data collection. In short, data for the process evaluation of child-centred PAR will be collected in T1, T2 and T4 through: (focus group) interviews with child co-researchers; reflection forms completed by child co-researchers; facilitators’ logbooks and reflection forms and interviews with facilitators. Data for the process evaluation of the facilitation, implementation and maintenance of co-created actions will be collected in T1, T2, T4 and T5 through: facilitators’ logbooks and reflection forms; interviews with facilitators and interviews with relevant adult actors. Data for the effect evaluation will be collected at T3, T4 and T5 through: a pre–post questionnaire; interviews with relevant adult actors; and action specific measurements such as systematic observation if new facilities will be implemented and participant registration if new activities will be implemented. Online supplemental file 2 provides the evaluation questionnaire and online supplemental file 3 provides the interview guides for interviews with child co-researchers and adult actors. For the evaluation of the effects of the co-created actions on children’s active outdoor play and related dietary behaviours, the extended cohorts design will be applied, which is a longitudinal cohort design with adjacent age cohorts (including children of similar age).^63^

**Table 1:** Overview of outcome items, data sources and timepoints for data collection, per component

Outcome items	Data sources	Timepoint*
*Systems*—Identification of the context and its drivers of insufficient outdoor play and related unhealthy dietary behaviours at multiple levels of the system		
Main drivers of outdoor play and related dietary behaviours	Data produced during mapping sessions, that is, list of main factors, system maps, description of main drivers	T1–T2
Resources (persons)	Adult actors recruitment form	T1
	Adult actors interview	T1
	Child co-researcher recruitment form	T1
Acceptability	Child co-researchers interview	T4
	Questionnaire	T5
*User perspectives*—Identification of children who use the co-created actions/participate in the co-created actions perspectives on developed actions		
Expectations/ readiness	Child co-researchers interview	T1
*Participatory Action Research (PAR) process*—(child level) identification of (1) important promoting and inhibiting factors perceived by child co-researchers and (2) impacts from the process on the child co-researchers		
Satisfaction/ dissatisfaction	Child co-researchers interview	T2+T4
	Child co-researchers reflection form	T1–T4
	Facilitator’s logbook and reflection form	T1–T4
Empowerment	Child co-researchers interview	T2+T4
	Child co-researchers reflection form	T1–T4
	Facilitator’s logbook and reflection form	T1–T4
	Facilitator interview	T2+T4
*Effects*—Identification of desired outcomes among the 6- to 12-year-old children in the local neighbourhoods		
Outdoor play/ physical activity	Questionnaire	T3+T5
	Adult actors interview	T5
	Action-specific measurements*	T3+T4
Related dietary behaviour	Questionnaire	T3+T5
	Adult actors interview	T5
	Action-specific measurements*	T3+T4
Recruitment	Adult actors recruitment form	T1
	Child co-researchers recruitment form	T1
*Reach*—(1) Key actors for the co-creation process; (2) children whose behaviours we aim to benefit as users of actions/participants in actions		
Users/ generalisability	Questionnaire	T5
	Action-specific measurements*	T4
Engagement/ commitment	Adult actors interview	T1+T2+T4
*Adoption*—(Actor level) identification of how (1) the PAR process and (2) the implementation of co-created actions is adopted by adult actors		
Support	Adult actors interview	T2+T4
	Facilitator interview	T2+T4
Delivery	Facilitator’s logbook and reflection form	T1–T4
	Adult actors interview	T5
	Action-specific measurements*	T4
*Iterative implementation*—Identification of (1) the PAR process are facilitated as planned and (2) the co-created actions are implemented as intended		
Facilitation	Facilitator’s logbook and reflection form	T1–T4
	Facilitator interview	T2+T4
*Maintenance*—Identification of sustained organisation of (1) the participatory way of working and (2) the implemented actions		
Sustainability	Adult actors interview	T5

T1, at the start of the mapping phase; T2, immediately after the mapping phase; T3, before implementation of actions; T4, immediately after implementation of actions; T5, approximately 6 months after implementation of actions (see figure 1).

* Action-specific measures are used to measure the effects of the specific local actions on outdoor play and related dietary behaviour, and include the monitoring of the systems level of the implemented actions. Data sources depend on the action(s) implemented and could be systematic observation, on-site interviews, participant registration and questionnaires.

By implementing the SUPER-AIM evaluation framework in all five countries, the B-Challenged project will be evaluated consistently across countries, which ensures consistency in data collection and allows combining and comparing data across countries. Additionally, through the monthly reflection meetings among facilitators, the process of applying the child-centred PAR protocol in five different countries will be monitored and challenges and solutions will be discussed to optimise the process in each country.

#### Analyses

Following data cleaning and data processing, qualitative (eg, interviews and logbook) and quantitative (eg, questionnaires and evaluation forms) data will be analysed both at the local neighbourhood level as well as across the five neighbourhoods. Qualitative data will be analysed by two independent researchers using thematic analysis based on Braun and Clarke,^64^ for example, in MAXQDA, NVIVO. Quantitative data will be analysed using multilevel regression analyses, to adjust for clustering of data within schools and neighbourhoods, and adjusting for relevant confounders (eg, age, gender), for example, in SPSS, R. Interaction terms will be added to the regression models to explore how combinations of intersectional identities—age, gender, ethnicity—modify effects of the actions.

### Patient and public involvement

The core of the B-challenged project will be to collaborate with key relevant local societal actors throughout the research and innovation process, from mapping needs to co-create, implement and evaluate actions targeting the physical and social environments related to active outdoor play and related dietary behaviours. Relevant societal actors include children (9–12 years), parents, professionals working with children (eg, teachers, youth workers) and professionals having influence on children’s behaviour (eg, school directors, local policy makers). Child co-researchers and key adult actors will mainly contribute to the project by participating in separate sessions, and a small number of collaborative sessions will be organised, for example, to obtain a shared understanding of the drivers of children’s active outdoor play and to co-create systemic actions. As a result of this multi-actor collaboration, the implemented co-created actions will better fit the local context and the values, needs and expectations of the children and other relevant societal actors. Throughout this process, B-Challenged will hold the principle of equity as a common value, and take a broad intersectional approach, considering gender, ethnicity and age as reciprocally constructing phenomena that in turn shape complex social and health inequalities. For example, through inclusive recruitment strategies, meaningful engagement of participating child co-researchers and adult actors, and acknowledging structural power relations (racism, ageism, sexism) and how they manifest in social and health inequalities. Through the multi-actor collaboration including actors responsible for the built environment and those delivering community-based physical and social activities, the co-created actions will be in line with local strategic plans and feasible in their delivery, and therewith sustainable.

### Ethics and dissemination

Ethics approval was obtained for the child-centred PAR approach by the ethics committees of the five local research institutions: Amsterdam University Medical Centers, Medical Ethical Committee, the Netherlands (2024.0785); University of Southern Denmark, Research Ethics Committee, Denmark (24/34821); Ethics Committee of Clinical Research of Aragon (CEICA), Spain (PI24/437); Ethics Committee University of Bremen, Germany (2025.28); The Bioethics Committee of the Institute of Mother and Child, Poland (60/2024). Ethics approval for the implementation and evaluation of the co-created actions was obtained in Denmark (Research Ethics Committee of the University of Southern Denmark; 24/34821 amendment 20250617) and Spain (CEICA; PI24/437), and will be obtained in the Netherlands, Germany and Poland. Participation will be voluntary, except for when the project is part of the school curriculum, and participants can withdraw their participation at any time without consequences, in the child-centred PAR process and the evaluation. Information will be provided to participating children, their parents and adult actors by members of the local research team through attractive and accessible information (eg, brochures, videos, sessions), explaining the purpose and nature of involvement, data collection and confidentiality. Next, signed informed consent forms will be obtained from one of the parents/caregivers of the participating children and the participating adult actors. Additionally, assent will be obtained by a member of the local research team from participating children, through children’s signature on the informed consent form or their verbal confirmation of wanting to participate at the start of the first session.

To ensure that child co-researchers can meaningfully contribute to the project, in the development of the child-centred PAR protocol, a lot of attention will be given to child-friendly activities and language.^39^ Furthermore, context-specific approaches and methods will be applied that emphasise trust, confidentiality and cultural sensitivity. To stimulate equality in power and decision making, participatory methods that stimulate equal power and decision-making will be applied, for example, children coming up with agreements on how to work together and using dot voting to ensure that the voices of all (child and adult) participants are represented. Additionally, evaluation methods (eg, questionnaires, observation protocols) will be discussed with child co-researchers and pretested before implementation. Furthermore, reflection on ethical principles takes place throughout the process of child-centred PAR, according to the ICPHR (International Collaboration for Participatory Health Research) ethical principles and practice guide,^65^ including enabling children to meaningfully contribute to decision-making and focusing on working together to achieve a positive change. Reflection on ethical principles will be organised in various ways: (1) each facilitator will reflect on the sessions they facilitated using a standardised facilitators’ logbook with reflection form (see online supplemental file 4); (2) monthly facilitators’ reflection meetings will be organised with facilitators from all participating countries to discuss ethical challenges and potential solutions and (3) child co-researchers will reflect on the sessions they participated in using standardised reflection forms (see online supplemental file 5).

The project results will be disseminated among the scientific community, professionals in health and other relevant sectors (including policy) and children and other citizens, for example, through open-access peer-reviewed publications, conference presentations, infographics and webinars. Following the principle ‘as open as possible, as restricted as necessary, as soon as possible’, where possible, datasets will be made available through open science platforms including Open Science Framework (www.osf.nl) and Zenodo (www.zenodo.org). Furthermore, project results will be disseminated in the local contexts by the project partners. Local media outlets and social media platforms (eg, Facebook pages of participating schools) will be used to share key highlights of the project. Project partners will raise awareness of the B-Challenged project among the local population to facilitate the mobilisation of appropriate resources—both human and financial—for the co-creation of actions, implementation of co-created actions and long-term maintenance of these actions.

## Discussion

The B-Challenged project aims to improve children’s active outdoor play and related dietary behaviours through co-creating physical and social environmental actions by combining child-centred PAR with methods from systems dynamics in underserved urban neighbourhoods. By involving relevant adult actors across various sectors (eg, education, urban planning, social services, sports) and children themselves, important information will be obtained on (1) the drivers of children’s active outdoor play and related dietary behaviours and (2) actions that can change the system in such a way that it stimulates these behaviours. Although actions may not be aligned with everyone’s interests, by collaborating with relevant adult actors and children themselves actions will be in line with the needs and wishes of relevant key actors and, therewith, potentially more sustainable and effective.

The B-Challenged project proposes a theory-based, child-friendly and structured child-centred PAR approach that provides rich data on multi-actor perspectives. The protocol for conducting child-centred PAR was based on the YoPA co-creation protocol, which was developed for conducting PAR with 12- to 19-year-old adolescents and other key actors in diverse, underserved urban environments in Europe and Africa.^39^ The YoPA protocol, in turn, was built on the LIKE (Lifestyle Innovations based on youth’s Knowledge and Experience) project, in which PAR and systems dynamics were applied in each step of the Intervention Mapping protocol with 10- to 14-year-old adolescents in Amsterdam, the Netherlands.^66^ The LIKE protocol proved successful in co-creating actions targeting different levels of the system together with adolescents; for example, adolescents developed a parenting evening about youth’s sleeping behaviour (ie, structures) and participated in decision-making about the design and organisation of public outdoor spaces (ie, goals).^32 67^ However, translating systems dynamic-related theory into practical activities in the participatory sessions was considered challenging and took a lot of time (ie, 3–4 school years).^66^ Moreover, in LIKE, there was suboptimal collaboration between the adolescent co-creators and adult co-creators/actors. Therefore, after developing the theoretical outline of the YoPA co-creation protocol, considerable attention was given to developing a practical, youth-friendly protocol, including a child-friendly vocabulary and integrating the contribution of adult actors in the youth-centred co-creation process.^39^ For the B-Challenged project, we aimed to minimise the number of participatory sessions (with children and adult actors) with a view to increase the feasibility of upscaling the child-centred PAR approach in future research projects. Minimisation of the number of participatory sessions in B-Challenged will be achieved by prescribing the activities in the sessions in more detail. As a consequence, the child co-researchers in B-Challenged will be less involved in decision-making regarding the design of the research process and the participatory sessions, for example, instead of letting children entirely decide on the research methods themselves, child co-researchers will be presented with a limited number of options they can choose from.

Despite a relatively short process when compared with the similar LIKE project,^66^ applying PAR with multiple key actor groups will be time and resources intensive, and may pose a number of logistical and relational challenges, including maintaining engagement and balancing power dynamics. By evaluating the process of applying the child-centred PAR approach in five different countries, important insights will be gathered on aspects of the participatory process that ‘worked’ in all countries, and those that needed translation to the local political, social and cultural context. These insights facilitate the application of multi-actor, child-centred PAR approaches in future projects in different contexts.

### Strengths and limitations

A strength of the B-Challenged project is the integration of evidence on the long-term effects of hypothetical environmental changes on health behaviours from analyses of existing cohort data in the mapping process. The child-centred PAR approach combined with methods from system dynamics is another strength of this project, taking into account the complexity of the various, multidimensional factors that drive children’s health-related behaviours as well as the perspectives of adult actors. By applying child-friendly methods and using child-friendly terminology, as developed in the YoPA protocol, participating child co-researchers (and adult actors) will be enabled to understand concepts and actively collaborate in methods from systems dynamics, for example, building a system map, identifying leverage points. However, aiming to include both children and adult actors throughout the PAR process—who participate on a voluntary basis—poses the risk of children and adult actors declining to participate or dropping out. By adapting the process to their needs, individual or job-related goals and schedules, we intend to promote that the project fits their everyday reality. The relatively short-term follow-up in the evaluation is a limitation of the project, as it is likely too short to achieve changes in the local system. Yet, by involving key adult actors including school staff and policy makers, our goal is to at least initiate actions at various levels of the local system that are sustained over time. Additionally, measuring maintenance—for example, of the co-creation process and co-created actions—will be limited to the relatively short-term follow-up. Nevertheless, interviews with adult actors 6 months after the implementation of actions might provide important insights into organisational changes that can support maintenance. Another limitation is the reliance on self-reported data for the effect evaluation, which is subject to recall and social desirability bias. Finally, the selected local neighbourhoods likely do not represent all underserved neighbourhoods and therefore, the findings of the project cannot be generalised to all underserved neighbourhoods in the participating countries.

## Supplementary material

10.1136/bmjopen-2025-108281 online supplemental file 1
