# Supplementary material for: Promoting active outdoor play and healthy dietary behaviours through the co-creation of supporting physical and social environments for and with primary school-aged children living in underserved neighbourhoods in Europe: the protocol of the B-Challenged project
Source: BMJ Open. 2026 Mar 6;16(3):e108281. doi: 10.1136/bmjopen-2025-108281 (PMC12970113; doi:10.1136/bmjopen-2025-108281)
Supplement: online supplemental file 1 [file bmjopen-16-3-s001.pdf]

SUPPLEMENTARY MATERIAL

Supplementary File 1. Details on selected neighbourhoods and recruitment procedures in each participating country.

Supplementary File 2. Questionnaire for effect evaluation.

Supplementary File 3. Interview guide for child co-researcher and adult actor interviews

Supplementary File 4. Facilitators' logbook including reflection form.

Supplementary File 5. Children's reflection form.

Supplementary File 1. Details on selected neighbourhoods and recruitment procedures in each participating country.

Country	Description of selected neighbourhood	Procedures for recruitment of children
**Denmark**	A neighbourhood in the city of Rudkøbing, on an Island called Langeland, was selected by the Danish research team.<br>The municipality of Langeland has approximately 12.000 inhabitants, which is making it one of the smallest municipalities in Denmark. It is located on the outskirts of Denmark and is sparsely populated, with only 41 inhabitants per square kilometre. The surrounding landscape is predominantly rural, featuring extensive green nature areas and being almost entirely encircled by water. The area face demographic shifts such as population decline and aging, where approximately 12% are children aged 0–17.<br>Additionally, the municipality struggles with socioeconomic challenges, including higher unemployment and lower educational levels than the national average.	Children were selected through a school in the neighbourhood by the PI, based on the sociodemographic characteristics of the neighbourhood. First, the PI contacted the school, which accepted the invitation to participate. Subsequently, Year 5 class (i.e., children aged 9-10 years) was selected by the school principle to participate in the project, as part of their school curriculum.
**Germany**	A neighbourhood in the northern part of the city of Bremen, at the edge of the city to a more rural area, was selected by the German research team. This neighbourhood has about 10. 000 residents. The neighbourhood was many established in the 1960ies with many housing block (mostly 5-story blocks), but also semi-detached houses. Apart from a couple of main streets, there is not too much traffic and it is quite greenish (tree from the 1960ies, large park nearby).<br>The sociodemographic make-up is mixed with migrants from Eastern Europe (Poland and former USSR) arriving in the 1990ies and a recent influx of refugees. Prevalence of childhood overweight/obesity (age 5-6, school entry) was	Children were selected through a school in the neighbourhood by the PI, based on based on the sociodemographic characteristics of the neighbourhood and the childhood overweight/obesity prevalence. Contact was made via a health educator at the school. Subsequently, a Year 3 class (i.e., children aged 8-9 years) was selected by the school headmaster to participate in the project during class time.

	19.9% in 2020-2022. Since 2020 the neighbourhood is part of a city programme for underserved neighbourhoods.	
**Poland**	A neighbourhood in the right-bank district of Warsaw was selected by the Polish research team. The with about 59,000 residents on 11.3 km² and a population density of around 5,500 people per km². The area combines pre-war tenements, post-war blocks, and revitalised industrial sites. Regeneration is ongoing, but social vulnerability remains high. The population is diverse: long-term working-class families, artists, young professionals, and migrants from Eastern Europe, mainly Ukraine and Georgia. Green space is limited - about 9.5 m² per resident, with green areas covering 5% of the district. There are 37 ha of parks, 12 ha of small greens, and 56 ha of street greenery. Key assets include Park Praski (18.5 ha), the Warsaw Zoo, and new pocket parks and biocenotic gardens. Recent greening efforts added over 8,800 shrubs and 113 trees. Despite improvements, the neighbourhood remains one of Warsaw's most socially vulnerable districts, with high unemployment intensity (32 per 1,000 working-age residents).	Children were recruited through a local community day-care centre attended by children up to 12 years of age from the selected neighbourhood . After reviewing a list of clubs, day-care centre, and other facilities for children and youth, this daycare centre was selected because the same group of children regularly attends after-school activities there throughout the school year, and they come from three nearby schools. First, the PI contacted the daycare centre director, who, after a presentation of the project, accepted the invitation to participate. Subsequently, the research team prepared and distributed information leaflets to parents of children aged 9 to 12 (grades 3–6 of primary school) via the daycare centre staff. Parents who wished their children to participate provided consent to the daycare supervisors.
**Spain**	A neighbourhood in located on the left bank of the city of Zaragoza was selected by the Spanish research team. Zaragoza is a medium-sized city in northeastern Spain, with around 700,000 inhabitants. This neighbourhood has about 78.548 residents. This neighbourhood, with the second highest percentage of population and the fourth largest in terms of area (of the urban districts), is made up of different neighbourhoods and even includes an industrial estate. The neighbourhood is classified as middle-income area but presents lower average income and education levels	The Education and Public Health departments from the local government suggested potential schools and supported the contact process. The schools were selected based on their socio-demographic characteristics and their participation profile in health initiatives. First, the Education and Public Health departments and PI contacted the school, which accepted the invitation to participate. Subsequently, Year 5 class (i.e., children aged 9-10 years) was selected. The research team and class tutors meeting with the families to

	compared to the city as a whole. Its cultural diversity and social context make it relevant setting for addressing health inequalities. The sociodemographic make-up is mixed with migrants, 10.8% of its total inhabitants. 43.5% come from Europe, 25.8% from Africa, 23.6% from America and almost 7% from Asia. The most represented countries are Romania with 31.8%, Morocco with 8.9%, followed by Ecuador, China, Colombia and Senegal, with between 4% and 6%.<br>In terms of public transport, it has above-average accessibility to urban and interurban buses and the council's bicycle system. It has a good provision of green areas, similar to the city average.	invite them to participate in the project, as part of their school curriculum.
**The Netherlands**	A neighbourhood in the western part of Amsterdam was selected by the Dutch research team. The neighbourhood had 9,365 residents in 2025, with an average density of 4,198 addresses per km2. Around 87% of the houses are built between 1950 and 1975, and 77,4% are apartments. The sociodemographic make-up is mixed with 22% of native Dutch, 11% of migrants from Europe, and 67% of migrants from outside of Europe. Of the inhabitants aged 15-75, 42% has followed a practical education, 32,9% a medium level education, and 25,1% and theoretical education. The neighbourhood has one of the highest number of children with overweight and obesity of Amsterdam (28% in 2017), and therefore, it is a focus area of the Amsterdam Approach to Healthy Weight.	Children were selected through a school in the neighbourhood by the PI, based on the characteristics of the neighbourhood (see neighbourhood description) and having a contact person at the school. First, the PI presented the project to the teachers at the school, who accepted the invitation to participate. Subsequently, teachers selected children from grades 6, 7 and 8 (i.e., children aged between 9 and 12), based on their interest and participating in previous projects. Based on this selection, two groups of child co-researchers were formed.

Supplementary File 2. Questionnaire for effect evaluation.

Section 1: Information about you and your family

1. How do you identify yourself?

☐ As a boy
☐ As a girl
☐ As …. [open answer]

2. In which year were you born?

☐ 2020
☐ 2019
☐ 2018
☐ 2017
☐ 2016
☐ 2015
☐ 2014
☐ 2013
☐ 2012
☐ I don't know

If you don't know the year in which you were born, please answer the next question.

2A. How old are you?

☐ 5 years
☐ 6 years
☐ 7 years
☐ 8 years
☐ 9 years
☐ 10 years
☐ 11 years
☐ 12 years
☐ 13 years

2B. In which month is your birthday?

☐ January
☐ February
☐ March
☐ April
☐ May
☐ June
☐ July
☐ August
☐ September
☐ October

□ November
□ December
□ I don't know

3. What class are you in? [Adapt to own COUNTRY]
□ Grade 1
□ Grade 2
□ Grade 3
□ Grade 4
□ Grade 5
□ Grade 6

4. What language do you most often speak at home?
□ [Insert language OF RESIDENCE]
□ *
□ *
□ *
□ Another language (fill out):
*Each country should make a list of the five largest immigrant groups in their country.

5. All families are different (for example, not everyone lives with both their parents, sometimes people live with just one parent, or they have two homes or live with two families) and we would like to know about yours.
Please answer this question for the home where you live all or most of the time and tick the people who live there.

□ Mother
□ Father
□ Stepmother (or father's girlfriend/partner)
□ Stepfather (or mother's boyfriend/partner)
□ Someone or somewhere else (e.g. grandparents). Please write it down
____

Siblings: Please say how many brothers and sisters live here (including half, step or foster brothers and sisters). Please write in the number or write 0 (zero) if there are none). Please do not count yourself.

How many brothers?_____________
How many sisters?_______________

6A. Does your father have a job?
□ Yes
□ No
□ Don't know or see my father

6B. Does your mother have a job?
 Yes
 No
 Don't know or see my mother

Section 2: Physical activity

Physical activity is any activity that increases your heart rate and makes you get out of breath some of the time. Physical activity can be done in sports, school activities, playing with friends, or walking to school. Some examples of physical activity are running, brisk walking, rollerblading,
biking, dancing, skateboarding, swimming, soccer, basketball, football and surfing [country-specific examples can be given].

7. Think about yesterday… (OBS not a weekend day)

	0 hours	0-½ hour	½-1 hour	1-1 ½ hour	1 ½-2 hours	> 2 hours
…How much time did you spend on physically active travel enough to raise your breathing rate? (walking, biking skateboarding – NO electric bike)						
…How much time did you do **organised** sports or other activities in which you were active enough to raise your breathing rate? (provide local examples in pictures soccer, dance club, fitness club)						
…How much time did you do **outdoor activities** in which you were active enough to raise your breathing rate? (provide local examples in pictures e.g. play at a playground, play soccer in the parc, skateboarding)						

7A. (If outdoor activities) With whom did you do outdoor activities?
 Friends
 Family members
 Alone

7B. Was yesterday a normal weekday?
 Yes
 No ___________________________
 I don't know

8. Think about last Sunday…

	0 hours	0-½ hour	½-1 hour	1-1 ½ hour	1 ½-2 hours	> 2 hours
…How much time did you spend on physically active travel enough to raise your breathing rate? (walking, biking skateboarding – NO electric bike)						
…How much time did you do **organised** sports or other activities in which you were active enough to raise your breathing rate? (provide local examples in pictures soccer, dance club, fitness club)						
…How much time did you do **outdoor activities** in which you were active enough to raise your breathing rate? (provide local examples in pictures e.g. play at a playground, play soccer in the parc, skateboarding)						

8A. (If outdoor activities) With whom did you do outdoor activities?
 Friends
 Family members
 Alone

8B. Was Sunday a normal weekend day?
 Yes
 No ________________________________
 I don't know

Section 3: Outdoor play

9. What is your perceived possibilities for playing outdoors in your neighborhood in…:

	Very good	Good	Neither or	Bad	Very bad
Parks					
Playgrounds					
Sport courts					
Streets					

10. How many times a week do you usually do following activities outdoors... ?

Please tick one circle for each line.

	Never	Less than once a week	Once a week	2-4 days a week	5-6 days a week	Once a day, every day	Every day, more than once
…Playing							
…Jogging							
…Skateboarding, roller skating etc.							
…Practicing sports							
…Biking							
… Water activities (swimming, rowing, surfing)							
…Hanging out							
…Other activities (e.g., scout)							

11. How many times a week do you usually do outdoor activities here... ?

Please tick one circle for each line.

	Never	Less than once a week	Once a week	2-4 days a week	5-6 days a week	Once a day, every day	Every day, more than once
…Street							
…Sport facilities							
…Equipped playground							
…Garden							
…Park							
…Nature area (e.g., forest, meadow)							
…Water area (e.g., lake, sea, shore, stream, river)							

Section 4: Dietary behaviour

12. How many times a week do you usually eat or drink following when you do activities outdoors ... ?
Please tick one circle for each line.

	Never	Less than once a week	Once a week	2-4 days a week	5-6 days a week	Once a day, every day	Every day, more than once
…Fruits							
…Vegetables							
…Sweets (candy or chocolate)							
…Crisps and other salty snacks							
…Coke or other soft drinks that contain sugar							
…Fast-food (pizza, kebab, shawarma, burgers)							
…Water							
…Energy drinks such as Red bull, Monster							

Section 5: Knowledge and usage of action(s) (ONLY follow-up)

13. Have you heard about…?

[Insert name /description of country specific actions - One row for each action]	Yes	No	Don't know
-			
-			
-			

13A. If, yes, what do you think about…?

[Insert name /description of country specific actions - One row for each action]	Very good	Good	Neither or	Bad	Very bad
-					
-					
-					

14. Have you tried…?

[Insert name /description of country specific actions - One row for each action]	Yes	No	Don’t know
-			
-			
-			

14A. If yes, how often do you…?

[Insert how to use the country specific action(s) tried - One row for each action]	Every day	4 to 6 times a week	2 to 3 times a week	Once a week	Once a month	Less than once a month
-						
-						
-						

Section 6: Country specific optional additional questions

Space for optional questions. Example of questions below:

Family Affluence Scale (FAS)

Description: a six-item measure of material family wealth as an alternative indicator of socioeconomic status, given the difficulties in obtaining reliable information on parental occupation.

Does your family own a car, van or truck?
☐ No
☐ Yes, one
☐ Yes, two or more

Do you have your own bedroom for yourself?
☐ No
☐ Yes

How many computers do your family own (including laptops and tablets, not including game consoles and smartphones)?
☐ None
☐ One
☐ Two
☐ More than two

How many bathrooms (room with a bath/shower or both) are in your home?
☐ None
☐ One
☐ Two
☐ More than two

Does your family have a dishwasher at home?
☐ No
☐ Yes

How many times did you and your family travel out of [insert country here] for a holiday/vacation last year?
☐ Not at all
☐ Once
☐ Twice
☐ More than twice

Sleep behaviour

In the past 7 days…

On average, what time did you go to sleep (lights, books, devices etc. off) in weekdays? __________

On average, what time did you wake up on weekdays?
____

On average, what time did you go to sleep (lights, books, devices etc. off) in weekend days?
____

On average, what time did you wake up on weekend days?
____

On which days did you feel sleepy during the day?
☐ Monday
☐ Tuesday
☐ Wednesday
☐ Thursday
☐ Friday
☐ Saturday
☐ Sunday

Screen time behavior

Think about yesterday, how much time did you spend on a computer, television, tablet, smartphone, or other electronic devices? (OBS not a weekend day)
☐ 0 hours
☐ 0-½ hour
☐ ½-1 hour
☐ 1-1½ hour
☐ 1½-2 hours
☐ More than 2 hours

Think about last Sunday… how much time did you spend on a computer, television, tablet, smartphone, or other electronic devices?

☐ 0 hours
☐ 0-½ hour
☐ ½-1 hour
☐ 1-1½ hour
☐ 1½-2 hours
☐ More than 2 hours

Supplementary File 3. Interview guide for child co-researcher (A) and adult actor (B) interviews.

A. Interview guide child co-researcher interviews

First interview – in the beginning of the mapping phase

Theme	Research question	Prompting questions
Intro	• Introduction	• Explain purpose of the interview<br>• Explain that there are no right or wrong answers and that they do not need to agree<br>• Explain that the interviewed person will be anonymized and can withdraw at any time
Readiness	• Does the project have a clear purpose for all child co-researchers?	• What do you know about the B-Challenged project?<br>• Are you in doubt of something about the project you would like to know more about?
	• Are the child co-researchers prepared to invest energy and work in the project?	• Do you feel ready to take part in the project?<br>- Why/why not?<br>• How will you react if you need to do a task you have not done before?
Expectations	• Are the child co-researchers likely to think it is a good idea?	• What do you think about the project?<br>- Why do you think that?<br>• Why do you want to take part in the project? (what motivates you)?<br>• What needs to be done/in focus for you to stay motivated?
	• What are the child co-researchers' expectations about their role in the project?	• What are you looking most forward to by taking part in this project?<br>- Why that?<br>• What do you think will become your most important task(s) in the project?<br>• What do you think about you being a researcher together with adults?<br>- Pros/cons?
	• Child co-researchers expectations to what benefits the intervention(s) will bring and to whom?	• Who do you think will get the most out of the project?<br>- Why them?<br>• Do you think the project will change things in your neighbourhood?<br>- If yes, why?<br>- If no, why not?

		• Do you think the project will change your outdoor activities or what you eat?<br>- If yes, why?<br>- If no, why not?
Outro	• Ending the interview	• Do you have anything you feel pertinent which we have not talked about?<br>• Do you have any questions about the interview or project in general?

Second and third interview– after the mapping phase and implementation phase

**Theme**	**Research question**	**Prompting questions**
Intro	• Introduction	• If you do not know each other, introduce yourself to each other<br>• Explain purpose of the study and interview<br>• Explain that there are no right or wrong answers and that they do not need to agree<br>• Explain that the interviewed person will be anonymized and can withdraw at any time
Satisfaction/Dissatisfaction	• Do the child co-researchers have negative or positive feelings to the project?	• How did you experience your participation in the project (so far)?<br>• What do you like most by taking part in this project? Why?<br>• What do you like least by taking part in this project? Why?<br>• What have been most challenging? Why?
	• Are the child co-researchers enthusiastically attached to the project?	• What motivates/motivated you to take part in this project?<br>• *Only interview 2:* What needs to be done/in focus for you to stay motivated?<br>• *Only interview 2:* Do you want to continue taking part in the project?<br>- If yes, why?<br>- If no, why not?<br>• *Only interview 3:* Has your motivation for taking part changed over time? If yes, can you explain how?<br>• Do you believe that what you do/did in this project will make a difference?<br>- If yes, how and for whom?<br>- If no, why not? How can this be changed?

Enabling environment	• Do the child co-researchers experience an enabling environment (resources, adult facilitation, sense-making, capacity-building)?	• Do you feel supported as a co-researcher?<br>- If yes- by whom?<br>- If not- what support do you need?<br>• Do you think it is fun to participate in this project?<br>- If yes- why?<br>- If not- why not?<br>• Do you feel safe (in the co-creation sessions) to express your opinion?<br>- If yes, please explain (why/how)?<br>- If no, – Why not?<br>• Do you feel listened to by the other children and adults in the project? (incl. trust/respect)<br>• What does it means to you to be listened to?<br>• Do you think you do activities in the project that were meaningful (e.g., conversations with adult-actors with power; activities in the community to get insight in e.g., the problem or solution)?<br>- If yes, please explain (why/how)?<br>- If no, – Why not?<br>• Have you been involved in taking decisions?<br>- If yes, please explain (why/how)?<br>- If no– Why not?<br>• Can you mention something you have learnt in the project? (e.g., skills, knowledge, critical thinking)<br>• Do you feel ownership to the project/intervention(s)?<br>- If yes, please explain (why/how)?
Empowerment	• Have the child co-researchers achieved empowerment (positive child development and participatory competences)?	• Can you describe a situation where you master a skill you did not believe you could master? (E.g., critical thinking, research skills, presenting, collaborating, making decision (e.g., related to healthy lifestyle behaviours)<br>• Have you experienced to be more self-confident/competent (e.g., in relation to change your health behaviour)?<br>- If yes, please explain (why/how)?

		- If no, – Why not?
Acceptability*	• Will child co-researchers see the point of the intervention(s)?	[mention the country specific action(s)]<br>• Are the implemented actions in line with how you as co-researchers developed them?<br>- If yes, why?<br>- If no, why not?<br>• What do you think about the actions made in this project? E.g. are they important?<br>- If yes, why?<br>- If no, why not?
	• Will child co-researchers use the intervention(s)?	• Have you made use or participate in any of the actions developed in this project [mention the country specific intervention(s)]?<br>- If yes, please explain how, how much<br>- If no, please explain what is needed for you to make use of the actions?<br>• In what way have the actions influenced what you are doing outdoors?<br>• In what way have the actions influenced what you are eating/buying of food?
Outro	• Ending the interview	• Do you have anything you feel pertinent which we have not talked about?<br>• Do you have any questions about the interview or project in general?

*Only ask questions related to acceptability in the third child co-researcher interview (i.e., after the implementation phase)

B. Interview guide adult actor interviews

First interview – at recruitment

**Introduction**<br>– Brief description of the project.<br><br>**Role, tasks, and responsibilities**<br>1. Can you describe your current role and your main responsibilities within your organization?<br>2. What specific tasks do you undertake that relate to children's outdoor play and related dietary behaviours?<br><br>**Perspectives on outdoor play and related dietary behaviours**

3. How do you view the importance of outdoor play for children? And related dietary behaviours?

Current contribution to the topic

4. How does your organization currently contribute to addressing the lack of outdoor play and related dietary behaviours among children?

Interest in the topic

5. How interested are you or your organization in addressing the issues of children's outdoor play and related dietary behaviours?
6. What motivates your interest in this topic?

Perceived level of power to address the topic

7. How would you describe your or your organization's ability to influence children's outdoor play and related dietary behaviours?

Potential future contributions to the topic

8. How could you contribute to improving children's outdoor play and related dietary behaviours moving forward?
9. How interested are you to be involved in future actions to move this forward?

Snowballing other actors

10. Can you recommend any specific actors or groups who have a significant impact on children's outdoor play and related dietary behaviours?

Second and third interview – after the mapping phase and after the implementation

**Theme**	**Research question**	**Prompting questions**
Intro	• Introduction	• If you do not know each other, introduce yourself to each other<br>• Explain purpose of the study and interview<br>• Explain that there are no right or wrong answers<br>• Explain that the interviewed person will be anonymized and can withdraw at any time
Engagement/Commitment	• Do the adult-actors feel involved and listened to?	• How have you experienced taking part in this project?<br>• What do you think about co-creation with children, in general<br>- Pros/cons

		• What do you think about the way adult-actors are being involved in this project?<br>- Pros/cons
	• Are the adult-actors enthusiastically attached to the project and continually works to achieve the sessions'/project's goal?	• What motivates you to take part in this project?<br>• *Only interview 2:* What needs to be done to keep you motivated?<br>• *Only interview 2:* Do you want to continue taking part in the project? Being part of the network?<br>- If yes – why?<br>- If no – why not?<br>• Has your motivation for taking part changed over time? If yes, can you explain how?<br>• What is your motivation for continuing taking part in an adult actor network after the project ends?
	• Do the adult-actors work with a sense of confidence that they are making a difference?<br>• Do the adult-actors believe that the co-created actions are important to implement?	• What do you think about the way your resources and/or knowledge have been brought into use?<br>• Could your resources and/or knowledge have been used better?<br>- If yes, how?<br>• Do you believe that you are making a difference by taking part in this project?<br>- If yes, in what way? Examples<br>- If no, why not?<br>• *Only interview 3*: Regarding the action(s) you implement/support, do you think this (these) action(s) is (are) important to implement?<br>• *Only interview 3:* Have you implemented the intervention/actions as was intended?<br>- If yes - why?<br>- If no – why not?
Outro	• Ending the interview	• Do you have anything you feel pertinent which we have not talked about?

		• Do you have any questions about the interview or project in general?<br>• Explain what the interview will be used for (locally and across countries)

Fourth interview – six month after the implementation phase

**Theme**	**Research question**	**Prompting question**
Intro	• Introduction	• If you do not know each other, introduce yourself to each other<br>• Explain purpose of the study and interview<br>• Explain that there are no right or wrong answers<br>• Explain that the interviewed person will be anonymized and can withdraw at any time
Perceived effects	• Whether adult-actor perceived any (positive) effects of the intervention(s) on children's outdoor play and related dietary behaviors?	• Do you perceive the intervention/s has/have an impact on children's outdoor play and/or related dietary behaviors?<br>- If yes, in what way? Examples<br>- If no, why not?
Sustainability	• Does the adult-actor still implement / support the implementation of the co-created actions, as intended or in adapted form?	• Have you continued to implement / support the implementation of the co-created actions (as intended)? ?<br>- If yes, what?<br>- If no, why not? What do you need in order to sustainably implement the intervention?<br>• Have you adapted the actions(s) to optimize implementation?<br>- If yes, how?<br>• Is there anything else that should be adapted or improved in the implementation to support sustainable implementation?<br>- If yes, what?
	• Has there been established any organizational agreements or partnerships for sustainable implementation?	• What has been done on an organizational level to support maintenance of intervention(s) implementation?<br>• What should be done forward-looking on an organizational level to better

		support maintenance of intervention(s)?<br>• What is your role in maintaining the intervention(s)?
	• Can the intervention(s) be adapted or improved on the basis of experience?	• How can the intervention(s) be adapted to be more impactful?<br>-
	• Have stakeholders implemented participatory approaches in their way of working	• Do you include children's voice in your usual practice now<br>- if yes, how do you do that?<br>- If not, why?<br>• Do you include children more or less in your usual practice now than prior being part of this project?<br>- If yes, why have your changed your practice?
Outro	• Ending the interview	• Do you have anything you feel pertinent which we have not talked about?<br>• Do you have any questions about the interview or project in general?<br>• Explain what the interview will be used for (locally and across countries)

Supplementary File 4. Facilitator's logbook including reflection form.
Based on previous work of Emke et al. [1] and the YoPA project [2].

**General session information**	
Phase: Participatory needs assessment (1) or Co-creation (2) (mark with a circle)	
Session number:	
Action team ID/number:	
Date:	
Start time:<br>End time:	
Facilitator(s) name(s):	
Session topic:	
Place for session:	
Session with child co-researchers ( ) with adult actors ( ) or both ( )	
Number of participants expected Nº ( ) and how many attended the session Nº ( )	
Reason for non-attendance or any dropout (if known):	
Were the session objectives achieved? (Yes) (No) (Partially)<br>Why? Please give more details:	
Were there any changes during the session concerning the original protocol?	
Were there any specific needs, unexpected insights or findings from this session?<br>If so, please give more details.	
Provide the detailed plan you prepared for the session and the specific activities (including energizers, capacity building, etc.). Indicate the name of the activity/ies in which the adaptations were made, **which** specific adaptations were made and **why** adaptations were needed.	
**Reflection**<br>- disagree, 0 neutral, + agree<br>(- = you don't agree with the statement, 0 = you neither agree or disagree with the statement or can't say anything about it, + = you agree with the statement)	
**Group process**	
The goal of the meeting was clear	(-,0,+)
Almost all participated actively in the meeting	(-,0,+)

Everyone could give their opinion	(-,0,+)
The atmosphere/vibe was good	(-,0,+)
The participants (child co-researchers and/or adult actors) could show their creativity	(-,0,+)
The children learned something new<br>[only fill out after sessions including children]	(-,0,+)
What went well?	Open question
Did the used participatory methods give the anticipated output?	Open question
Which qualities did arise within the group?	Open question
How can we promote the qualities within the group more?	Open question
What can be improved according to the group process?	Open question
Were all of the aims of the session reached? If not, why not? If yes, how where they reached?	Open question
To what extent were participants (child co-researchers and/or adult actors) in the lead during session? How did this become visible? How was this caused?	Open question
If children learned something new – what did they learn?<br>[only fill out after sessions including children]	Open question
**Role of the facilitator**	
I was clear	(-,0,+)
I involved everyone	(-,0,+)
I had a positive influence on the group atmosphere	(-,0,+)
What went well for you as facilitator?	Open question
Which personal qualities arose during the sessions?	Open question
How did your mood and personal characteristics influence the session?	Open question
What can be improved according to your role as a facilitator?	Open question

Supplementary File 5. Child co-researchers reflection form

Based on previous work of Emke et al. [1] and the YoPA project [2].

**What did you think of this meeting?**					
	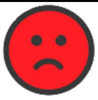	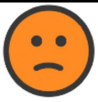	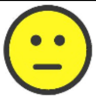	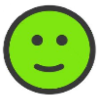	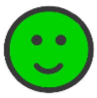
It was fun					
It was clear what we were going to do					
The meeting was useful					
I participated well					
I thought it was easy to say what I think					
I thought the atmosphere was good					
I have been creative					
I learned something new					
I look forward to the next meeting					
What did you think went well today?					
What did you learn today?					
What was most difficult today?<br>And how did you manage?					
What do you think we should do differently next time?					
What can the facilitators do better next time?					
Is there anything else you want to say about today's meeting?					

REFERENCES

1. Emke H, Vandendriessche A, Chinapaw M, Deforche B, Verloigne M, Altenburg T, et al. Facilitating co-research: lessons learned from reflection forms within three participatory action research projects. Health Res Policy Syst. 2024;22(1):117.
2. Chinapaw MJM, Klaufus LH, Oyeyemi AL, Draper C, Palmeira AL, Silva MN, et al. Youth-centred participatory action approach towards co-created implementation of socially and physically activating environmental interventions in Africa and Europe: the YoPA project study protocol. BMJ Open. 2024;14(2):e084657.
