# Supplementary material for: Promoting active outdoor play and healthy dietary behaviours through the co-creation of supporting physical and social environments for and with primary school-aged children living in underserved neighbourhoods in Europe: the protocol of the B-Challenged project
Source: BMJ Open. 2026 Mar 6;16(3):e108281. doi: 10.1136/bmjopen-2025-108281 (PMC12970113; doi:10.1136/bmjopen-2025-108281)
Supplement: Supplementary file 2 [file bmjopen-2025-108281.reviewer_comments.pdf]

PEER REVIEW HISTORY

BMJ Open publishes all reviews undertaken for accepted manuscripts. Reviewers are asked to complete a checklist review form and are provided with free text boxes to elaborate on their assessment. These free text comments are reproduced below.

ARTICLE DETAILS

Title (Provisional)

Promoting active outdoor play and healthy dietary behaviours through the co-creation of supporting physical and social environments for and with children: the B-Challenged project

Authors

Altenburg, Teatske M; Pawlowski, Charlotte S; Ahrens, Wolfgang; Brand, Tilman; Buck, Christoph; Börnhorst, C; Dzielska, Anna; Klaufus, Leonie; De Miguel-Etayo, Pilar; Moreno, Luis; Okulicz-Kozaryn, Katarzyna; Vrijkotte, Tanja G. M.; Wink, Renée; Belmon, Laura S; Chinapaw, Mai

---

VERSION 1 - REVIEW

**Reviewer**	**1**
**Name**	**Boggiano, Victoria**
**Affiliation**	**University of North Carolina System, Family Medicine**
**Date**	**19-Nov-2025**
**COI**	**None**

Overall, this is an interesting concept tackling an important topic - children's access to and utilization of green spaces and healthy foods to help meet goals for physical activity and dietary behaviors. I really like what B-challenged is setting out to do. There are several parts of the manuscript that could be edited for clarity about the study design and protocol, as detailed below.

Introduction:

- Which public health guidelines are you referring to in paragraph 1? I would specify in addition to including in references. In that same sentence, please specify which region you are referring to.

- 15% of 11-15 year old adolescents consuming a sugar sweetened beverage does not sound like a very high number (though we strive to have it as low as possible!), please clarify.

- Please define early on in the introduction what you mean by lower income and marginalized groups which is used throughout the rest of the introduction

- Paragraph two, please clarify what you mean by underserved neighborhoods.

- Consider rephrasing "their mothers are more hesitant to stimulate their children's outdoor plan"

- Consider rephrasing "have a high responsiveness to external food cues and experience difficulties with matching intake of energy needs" - I'm not sure what you mean here.

- I would avoid the phrase "children's unhealthy lifestyle behaviors" since it is phrased in such a way that it may seem to assign blame

- What is "systems dynamics" referenced in the fourth paragraph?

- Interesting comment around safety of neighborhoods, as often children perceive neighborhoods as unsafe because they ARE unsafe. Did you find any exploration about this in the literature?

- Consider explaining in the introduction why these five European countries were chosen - do these countries face higher burdens of barriers to healthy lifestyles for children?

- Consider clarifying either here or early in the methods section what the current state of these local neighborhoods is in the five countries chosen, if this data exists. How often in the present state do children have green space to play in? How often do they have access to healthy foods?

- The last paragraph of the introduction could be more clear. What types of health behaviors are you assessing and what do you hope the results will help to do in each of the five countries? What types of systems change are you interested in evaluating? Please revise the end of the introduction for clarity.

Methods:

- As above, consider specifying why these five countries were chosen.

- It is not clear what the co-creation protocol means when used early on in the methods section. Please define more clearly in the study design section. For instance, how are children taught how to conduct research? How are they taught to analyse collected data?

- It is also not clear early on what the systems levels are that you are referring to, which is relevant for later in the methods section.

- In the settings and participants section, what evidence do you have that you can cite that most children use pocket money to buy unhealthy foods? I would suggest revising language so as not to cite blame (i.e. perhaps only unhealthy foods are available for purchase?)

- Excellent description here of what constitutes an underserved neighborhood. Please cite a reference or references for this section.

- There is lots of switching of age ranges (6-12 vs 9-12)- please revise for consistency.

- You mention recruiting from underserved neighborhoods in five countries but you do not specify how local neighborhoods are chosen or how many local neighborhoods are chosen in each country. Please specify.

- How is a general list of potentially relevant adult actors initially generated? (See "Key adult actors" section)

- Who does the interviews with the identified adult actors and how are adults selected? Current description is vague, more detail would be helpful.

- How are children selected from the local schools?

- Given the diversity of settings in all five countries that this will be conducted in, how is fidelity to the study protocol maintained?

- Please clarify why you are using the IDEFICS and ABCD studies here and what you are hoping to use them for. Also, please clarify why these studies are relevant to your work given that the age ranges and countries are not exactly the same as some in your study population.

- Ethics and dissemination: If the study is ongoing since 2024, where has ethical approval already been obtained? What study elements have been ongoing since 2024?

- How is informed consent obtained from families, i.e. who obtains it from participants?

- How and when are questionnaires provided for the quantitative part of the evaluation? When are interviews conducted for the qualitative part of the evaluation (I do see this in Table 1 but it would be good to briefly mention this in the methods section as well).

Discussion:

- The first paragraph could be simplified and edited for clarity.

- It would be helpful either in the discussion or earlier to describe more about the YoPA protocol and the LIKE protocol, as it is still hard to conceptualize exactly what is meant by PAR and systems dynamics. Some clear examples would be helpful.

- Similarly, either in the discussion or earlier, more clarity on how children are taught research skills and their interactions with the study team and the adult actors would be helpful. It is not clear what is meant by the last sentence of the second paragraph that child co-researchers will have "less decision-making power regarding the design of the co-creation sessions" and how this differs from YoPA and LIKE etc.

- In the strengths and limitations section, I suggest adding that the local neighborhoods chosen in these five countries may not represent all underserved communities in each of the countries or Europe as a whole.

- Given that this study has been ongoing since 2024, some baseline data or updates on recruitment to date in this section might be helpful if possible.

VERSION 1 - AUTHOR RESPONSE

Reviewer: 1
Miss Victoria Boggiano, University of North Carolina System
Comments to the Author:

Overall, this is an interesting concept tackling an important topic - children's access to and utilization of green spaces and healthy foods to help meet goals for physical activity and dietary behaviors. I really like what B-challenged is setting out to do. There are several parts of the manuscript that could be edited for clarity about the study design and protocol, as detailed below.

REPLY

We thank the reviewer for acknowledging the importance of our B-Challenged study and appreciate the comments/questions, which were very helpful to further improve our manuscript.

Introduction:

- Which public health guidelines are you referring to in paragraph 1? I would specify in addition to including in references. In that same sentence, please specify which region you are referring to.

REPLY

With public health guidelines we refer to guidelines on physical activity and healthy diet for adolescents in the European region. We acknowledge that although physical activity recommendations are global, recommendations for healthy diet are regional or national. We have rephased the first sentence of our introduction (page 5, lines 102-108):

"An alarmingly low number of children and adolescents meet public health guidelines on physical activity and healthy dietary behaviours: only 7-33% of 5-to-17-year-old children and adolescents globally are sufficiently active (1), 4-52% of European children and adolescents meet European guidelines for fruit and vegetable intake (2-4) and, for example, more than half of 12-17-year-old German adolescents do not achieve German food-based dietary recommendations for fruits and vegetables, and consume far above the recommended amounts of unfavourable foods such as confectionary (5)."

- 15% of 11-15 year old adolescents consuming a sugar sweetened beverage does not sound like a very high number (though we strive to have it as low as possible!), please clarify.

REPLY

We agree with the reviewer that 15% of adolescents consuming sugar sweetened beverages does not sound like an alarmingly high number – despite European recommendations to limit the intake of sugar-containing products (Food-Based Dietary Guidelines recommendations for sweets and desserts | Knowledge for policy). We have substituted this example with an example from a study among 1353 German adolescents (page 5, lines 105-108):

"… more than half of 12-17-year-old German adolescents do not achieve German food-based dietary recommendations for fruits and vegetables, and consume far above the recommended amounts of unfavourable foods such as confectionary (5)."

- Please define early on in the introduction what you mean by lower income and marginalized groups which is used throughout the rest of the introduction

REPLY

We apologize for not being clear and have rephrased this sentence as follows (page 5, lines 110-114): *"Importantly, this burden is disproportionally distributed across educational, employment and migrant groups, with children of parents with low/medium (vs. high) education, children with unemployed (vs. employed) parents and children with a migrant background (vs. native) being less likely to engage in favourable health-related behaviours resulting in higher proportion of* overweight *(7-9)."*

- Paragraph two, please clarify what you mean by underserved neighborhoods.

REPLY

We have moved the definition of what we mean by underserved neighbourhoods from the methods section to the introduction (page 5, lines 122-125):
"… neighbourhoods with a high percentage of families characterised by lower income, lower levels of education, more health disadvantages (e.g., physical/mental health problems, safety issues) and limited availability of attractive and safe (green) spaces for outdoor play."

- Consider rephrasing "their mothers are more hesitant to stimulate their children's outdoor play"

REPLY

We are not sure if we understood the reviewer's comment correctly and assume it is about 'mothers' being hesitant to stimulate their children's outdoor play. As both studies were conducted among mothers, we have not rephrased the sentence.
However, when checking the references, we noticed that one of the references was incorrect (i.e., Cohen et al, 2012, Soc Sci Med) and have replaced it with the correct one (i.e., Clements, 2004, Contemp Iss Early Ch).

- Consider rephrasing "have a high responsiveness to external food cues and experience difficulties with matching intake of energy needs" - I'm not sure what you mean here.

REPLY

We have clarified this sentence as follows (page 5-6, lines 134-136):
"… are sensitive to external food cues and experience difficulties with matching energy intake to physiological energy demands"

- I would avoid the phrase "children's unhealthy lifestyle behaviors" since it is phrased in such a way that it may seem to assign blame

REPLY

We have rephrased this as follows (page 6, line 139-140):
"promoting favourable health-related behaviours in children"

- What is "systems dynamics" referenced in the fourth paragraph?

REPLY

We have clarified what we mean with systems dynamics and added a reference. The sentence now reads (page 6, lines 145-150):
"Approaches from systems dynamics are aimed at tackling complex problems by addressing the dynamic nature and interconnectedness between the various factors within a system that drive complex problems (31), and are therefore increasingly used to obtain a better understanding of complex problems such as unfavourable health-related behaviours, and subsequently develop actions to change the system (32, 33)."

- Interesting comment around safety of neighborhoods, as often children perceive neighborhoods as unsafe because they ARE unsafe. Did you find any exploration about this in the literature?

REPLY

We agree it is interesting to verify children's experiences of their neighbourhood being unsafe. However, we believe that it is important to address children's perceptions of unsafety irrespective of whether these perceptions are correct, as both perceptions (e.g., of children/adolescents and their parents) and objective measures of unsafety are associated with children's physical activity (Carver et al, Health & Place, 2007). Therefore, we did not explore this in the literature.

- Consider explaining in the introduction why these five European countries were chosen - do these countries face higher burdens of barriers to healthy lifestyles for children?

REPLY

Thank you for this question. The five European countries were chosen in response to a call for proposals (i.e., these countries could apply for funding; ERA4Health HealthEquity call) and relevant expertise among and successful previous collaborations with partners in the included countries.

- Consider clarifying either here or early in the methods section what the current state of these local neighborhoods is in the five countries chosen, if this data exists. How often in the present state do children have green space to play in? How often do they have access to healthy foods?

REPLY

We agree that such information on the selected neighbourhoods would be valuable to present. We have provided descriptions regarding the neighbourhoods' sociodemographic and physical environmental characteristics in a new table (Table 1; page 21-23).
We have referred to this table in the text as follows (page 8-9, lines 240-242):
"Table 1 provides descriptions of the sociodemographic and physical environment of the selected neighbourhoods in each participating country."

- The last paragraph of the introduction could be more clear. What types of health behaviors are you assessing and what do you hope the results will help to do in each of the five countries? What types of systems change are you interested in evaluating? Please revise the end of the introduction for clarity.

REPLY

We apologize for not being fully clear about what we aim to achieve. First of all, with health behaviours we refer to active outdoor play and healthy dietary behaviours, as explained in sentences

168-169. We have added examples of the types of healthy dietary behaviours we aim to address (page 7, lines 175-176):
"With related healthy dietary behaviours we refer to children's consumption of water, fruits and vegetables right before, during or following their outdoor play."

Regarding systems changes, the type of changes we are interested in evaluating in the five countries depends on the mechanisms that drive children's active outdoor play and dietary behaviours. For example, in case no safe and/or attractive playgrounds are available in the selected neighbourhoods, we will advocate with municipalities and urban planners to develop playgrounds in collaboration with children. In this case, part of the system change we strive for could contain sustainable engagement of children perspectives in playground design by local policy makers and urban planners. To clarify this, we have added this example (page 7, lines 178-179):
"… if the co-created and implemented actions positively contributed to systems change (e.g., local policy makers and urban planners engage children in the development of attractive playgrounds)."

Methods:
- As above, consider specifying why these five countries were chosen.
REPLY
We kindly refer to our reply to the reviewer's previous comment regarding this point.

- It is not clear what the co-creation protocol means when used early on in the methods section.
REPLY
We have rearranged the study design section, now first explaining the two phases of the B-Challenged approach of child centred PAR with methods from systems dynamics and thereafter explaining the basis of this protocol.
We kindly refer to the study design paragraph on page 6-7, lines 185-224.

Please define more clearly in the study design section. For instance, how are children taught how to conduct research? How are they taught to analyse collected data?
REPLY
Conducting research and analysing collected data are examples of capacity building activities. These activities might vary between countries, e.g., depending of what children need and are learned by presenting various research methods and facilitate discussions. We have provided examples of capacity building activities (page 8, lines 212-216):
"Capacity building is an important aspect of the child-centred PAR approach, to enable child co-researchers to conduct research (e.g., research methods, privacy protection), basic analyses of collected data and present findings, for example, by presenting various research methods, facilitating discussions on how to protect privacy, demonstrating how to analyse data and practicing presentations."

- It is also not clear early on what the systems levels are that you are referring to, which is relevant for later in the methods section.
REPLY

We thank the reviewer for this comment and have added information in the methods section regarding the system dynamics methods (including system levels) that are incorporated in the protocol (page 8, lines 216-221):
"Additionally, and similar to the YoPA co-creation protocol (39), this protocol is based on methods from systems dynamics: 1) Group Model Building (41), to develop a shared understanding of the main factors and interrelations that drive children's active outdoor play and dietary behaviours in the local neighbourhoods, and 2) the Action Scales Model (42), to describe the four levels at which action can take place, with increasing impact on system change (i.e., events, structures, goals and beliefs). Facilitating researchers in each country apply the practical protocol to their local context."

- In the settings and participants section, what evidence do you have that you can cite that most children use pocket money to buy unhealthy foods? I would suggest revising language so as not to cite blame (i.e. perhaps only unhealthy foods are available for purchase?)

REPLY

We have added the references to two studies that showed that receiving pocket money allows children to buying unhealthy food. However, we realise that the wording in our methods section was too strong. We have rephrased this sentence as follows (page 9, lines 245-246):
"…, which allows them to buy unhealthy foods (16, 46)."
Full references:

– Li M, Xue H, Jia P, Zhao Y, Wang Z, Xu F, Wang Y. Pocket money, eating behaviors, and weight status among Chinese children: the childhood obesity study in China mega-cities. Prev Med. 2017;100:208–15
– Emke, H., Chrifou, R., Dijkstra, C. *et al.* A participatory needs assessment to map the system dynamics of overweight related behaviours of 10-14-year-old adolescents living in an underserved neighbourhood in Amsterdam, the Netherlands. *BMC Public Health* **25**, 324 (2025).

- Excellent description here of what constitutes an underserved neighborhood. Please cite a reference or references for this section.

REPLY

We thank the author for this compliment on this description, and have moved it to the introduction section, where we refer to underserved neighbourhoods for the first time. This definition is agreed-upon by the B-Challenged consortium, but not in the scientific literature. Therefore, we do not have a citation with this definition.

- There is lots of switching of age ranges (6-12 vs 9-12)- please revise for consistency.

REPLY

We apologize for not being clear about the different age ranges. The B-Challenged project is aimed at stimulating active outdoor play and healthy dietary behaviours in 6-12-year-old children. However, for co-creating and implementing actions, our protocol was designed for co-creation with children aged 9-12-year-old. Co-creation with younger children would have required a different approach which was not feasible in B-Challenged. Moreover, previous studies have shown that 9-12-year-old

children are very capable of taking the perspectives and needs of younger children into account (e.g. Caro et al).
We have clarified this as follows:

- *"The B-Challenged project aims to stimulate active outdoor play and healthy dietary behaviours in primary school-aged children – aged 6-12 years – living in underserved neighbourhoods in the five European countries."* (page 8, lines 234-236)
- *"In previous research we demonstrated that children from the age of 9 have an increased attention span, can more clearly see the viewpoint of others, are capable of participating in research projects, when using participatory age-appropriate methods, and are able to think for their younger peers when mapping needs and co-creating actions (47, 48). Therefore, in each local neighbourhood, 15-20 children aged 9-12 years are recruited to participate as co-researchers, representing 6-12-year-old children in their local neighbourhood."* (page 9, lines 253-258)

- You mention recruiting from underserved neighborhoods in five countries but you do not specify how local neighborhoods are chosen or how many local neighborhoods are chosen in each country. Please specify.
REPLY
We have added the following information to clarify the selection of neighbourhoods in each country (page 8, lines 237-240):
"In each country, one underserved neighbourhood is selected, based on our definition of having a high percentage of families characterised by lower income, lower levels of education, more health disadvantages (e.g., physical/mental health problems, safety issues) and limited availability of attractive and safe (green) spaces for outdoor play."

- How is a general list of potentially relevant adult actors initially generated? (See "Key adult actors" section)
REPLY
We have clarified this as follows (page 9, lines 267-273):
"In each local neighbourhood, a network of 15-20 local adult stakeholders, including parents, teachers, youth workers, academic researchers, urban planners, policy makers, is established through actor mapping (49, 50), which is conducted by 1-2 members of the local research team. First, researchers generate a list of potentially relevant actors across various sectors (e.g., education, urban planning, social services, sports), based on stakeholders/organisations situated in the local neighbourhood who/that are expected to hold important perspectives and position regarding children's outdoor play and dietary behaviours in the selected neighbourhoods."

- Who does the interviews with the identified adult actors and how are adults selected? Current description is vague, more detail would be helpful.
REPLY
Interviews with identified adult actors are done by 1-2 members of the local research team. We have added this information to the manuscript (page 9, line 269):
"…, which is conducted by 1-2 members of the local research team."

Additionally, we have provided more information on the process of actor mapping (page 9, lines 269-273):
"First, researchers generate a list of potentially relevant actors across various sectors (e.g., education, urban planning, social services, sports), based on actors/organisations situated in the local neighbourhood who/that are expected to hold important perspectives and position regarding children's outdoor play and dietary behaviours in the selected neighbourhoods."

- How are children selected from the local schools?

REPLY
The recruitment/selection of children in the selected neighbourhood varied between the countries. We have added information regarding these procedures for each included neighbourhood in a new table (i.e., Table 1). (Page 21-23)
We have referred to this table at page 8-9, lines 240-242:
"Table 1 provides a description of the process of selecting children for each selected neighbourhood."

- Given the diversity of settings in all five countries that this will be conducted in, how is fidelity to the study protocol maintained?

REPLY
We would like to clarify that the child-centred PAR protocol describes the general approach of applying child-centred PAR. The protocol includes proposed activities while allowing flexibility to adapt sessions to the local context, and child-friendly wordings of theoretical concepts. (pages 7-8, lines 205-208)
We organize monthly facilitation meetings to support facilitating researchers and align the child-centred PAR in the different countries (page 8, lines 222-224):
"Facilitating researchers in each country apply the practical protocol to their local context. Monthly reflection meetings are organized to support facilitators and align the child-centred PAR process across countries."

- Please clarify why you are using the IDEFICS and ABCD studies here and what you are hoping to use them for. Also, please clarify why these studies are relevant to your work given that the age ranges and countries are not exactly the same as some in your study population.

REPLY
We have rephrased the paragraph describing the use of the IDEFICS/I.Family and ABCD cohort studies to clarify the relevance and use of these cohort studies (page 10, lines 286-301):
"The main drivers of children's active outdoor play and related dietary behaviours are investigated through analysis of existing cohort data (phase 1A) and co-creation of local system maps together with child co-researchers and adult actors that visualise the main drivers of active outdoor play and related dietary behaviours (phase 1B). For the cohort analyses, data include physical and social environmental factors as well as area-level monitoring data linked to the IDEFICS/I.Family cohort (IDEFICS - Identification and prevention of Dietary- and lifestyle-induced health EFfects In Children and infantS; and I.Family study; (51)) and the ABCD (Amsterdam Born Children and their Development; (52)) cohort (phase 1A). The IDEFICS/I.Family and ABCD cohort studies are selected as

they include children from similar European countries as those included in the co-creation (in phase 1B and phase 2). Only waves including data of similarly aged children are considered in the analyses. The results from the cohort analysis provide insights into the causality and interconnectedness of environmental factors influencing health behaviours in children and adolescents from diverse European regions. Evidence will subsequently feed into the system maps that are developed in phase 1B. "

Ethics and dissemination:

-If the study is ongoing since 2024, where has ethical approval already been obtained? What study elements have been ongoing since 2024?

REPLY

As indicated on pages 15-16, lines 482-487, ethics approval is obtained for conducting child-centred PAR by the ethics committees of the five local research institutions. For the implementation and evaluation of the developed actions ethical approval is obtained in Denmark and Spain, and in progress in Germany, Poland and the Netherlands. We have added this information to the manuscript (page 16, lines 487-490):

"Ethics approval for the implementation and evaluation of the co-created actions is obtained in Denmark (Research Ethics Committee of the University of Southern Denmark; 24/34821 amendement 20250617) and Spain (Ethics Committee of Clinical Research of Aragon; PI24/437), and in progress in the Netherlands, Germany and Poland."

- How is informed consent obtained from families, i.e. who obtains it from participants?

REPLY

Thank you for this question. We have provided this information to the manuscript (page 16, lines 494-499):

"Members of the local research team provided information to participating children, their parents and adult actors through attractive and accessible information (e.g. brochures, videos, sessions), explaining the purpose and nature of involvement, data collection, and confidentiality. Next, they obtained signed informed consent forms from participating children, one of their parents/caregivers and adult actors."

- How and when are questionnaires provided for the quantitative part of the evaluation? When are interviews conducted for the qualitative part of the evaluation (I do see this in Table 1 but it would be good to briefly mention this in the methods section as well).

REPLY

We have added the following information to the text (page 14, lines 422-433):

"Table 2 provides an overview of the SUPER-AIM components, outcome items, data sources and timepoints for data collection. In short, data for the process evaluation of child-centred PAR is collected in T1, T2, and T4 through: (focus group) interviews with child co-researchers; reflection forms completed by child co-researchers; facilitators' logbooks and reflection forms; and interviews with facilitators. Data for the process evaluation of the facilitation, implementation and maintenance of co-created actions is collected in T1, T2, T4, and T5 through: facilitators' logbooks and reflection forms; interviews with facilitators; and interviews with relevant adult actors. Data for the effect

evaluation is collected at T3, T4 and T5 through: a pre-post questionnaire; interviews with relevant adult actors; and action specific measurements such as systematic observation if new facilities are implemented and participant registration if new activities are implemented. Supplementary Files 3 provides the evaluation questionnaire and Supplementary File 4 provides the interview guides for interviews with child co-researchers and adult actors."

Discussion:

- The first paragraph could be simplified and edited for clarity.

We agree that this paragraph could be simplified and have rephrased it as follows (page 17, lines 533-547):

"The B-Challenged project aims to improve children's active outdoor play and related dietary behaviours through co-creating physical and social environmental actions by combining child-centred PAR with methods from systems dynamics in underserved urban neighbourhoods. By involving relevant adult actors across various sectors (e.g., education, urban planning, social services, sports) and children themselves, the project will obtain important information on 1) the drivers of children's active outdoor play and related dietary behaviours and 2) actions that can change the system in such a way that it stimulates these behaviours. Collaboration with relevant adult actors and children themselves ensures that actions are in line with the local political, social and cultural contexts as well as the needs and wishes of relevant key actors, and therewith, potentially more sustainable and effective."

- It would be helpful either in the discussion or earlier to describe more about the YoPA protocol and the LIKE protocol, as it is still hard to conceptualize exactly what is meant by PAR and systems dynamics. Some clear examples would be helpful.

REPLY

In response to a previous comment of the reviewer, we have restructured the study design paragraph in the methods section and added more information on the PAR approach and methods from systems dynamics. We kindly refer to the study design paragraph on pages 7-8, lines 185-224. Additionally, we have provided an examples of actions developed in collaboration with adolescents in the discussion section (page 18, lines 557-559):

"… for example, adolescents developed a parenting evening about youth's sleeping behaviour (i.e., structures) and participated in decision-making about the design and organisation of public outdoor spaces (i.e., goals) (32, 67)."

- Similarly, either in the discussion or earlier, more clarity on how children are taught research skills and their interactions with the study team and the adult actors would be helpful.

REPLY

In response to a previous comment of the reviewer, we have provided more information on capacity building activities that are part of our child-centred PAR approach, including example activities (page 8, lines 212-216):

"Capacity building is an important aspect of the child-centred PAR approach, to enable child co-researchers to conduct research (e.g., research methods, privacy protection), basic analyses of collected data and present findings, for example, by presenting various research methods, facilitating

discussions on how to protect privacy, demonstrating how to analyse data and practicing presentations."

- It is not clear what is meant by the last sentence of the second paragraph that child co-researchers will have "less decision-making power regarding the design of the co-creation sessions" and how this differs from YoPA and LIKE etc.

REPLY

We have clarified the sentence which now reads (page 18, lines 569-573:

"As a consequence, the child co-researchers in B-Challenged are less involved in decision-making regarding the design of the research process and the participatory sessions, e.g., instead of letting children entirely decide on the research methods themselves, child co-researchers are presented with a limited number of options they can choose from."

- In the strengths and limitations section, I suggest adding that the local neighborhoods chosen in these five countries may not represent all underserved communities in each of the countries or Europe as a whole.

REPLY

We agree with this and have included the following limitation to the strengths and limitations paragraph (page 19, lines 604-607):

"Finally, the selected local neighbourhoods likely do not represent all underserved neighbourhoods and therefore, the findings of the project cannot be generalized to all underserved neighbourhoods in the participating countries."

- Given that this study has been ongoing since 2024, some baseline data or updates on recruitment to date in this section might be helpful if possible.

REPLY

We agree with the reviewer and have added a table (Table 1; page 21-23) providing descriptions regarding the neighbourhoods' sociodemographic and physical environmental characteristics as well as the process of recruiting children in each local neighbourhood.

VERSION 2 - REVIEW

**Reviewer**	**1**
**Name**	**Boggiano, Victoria**
**Affiliation**	**University of North Carolina System, Family Medicine**
**Date**	**29-Jan-2026**
**COI**	

The authors have further explained what the study outcome and purpose is, why the locations were chosen, and what the steps are in terms of recruitment of participants and

co-creation of knowledge to create more spaces and food options for children in the underserved regions of the five countries selected. I recommend publication of the study protocol.
